# Specific Antibody and the T-Cell Response Elicited by BNT162b2 Boosting After Two ChAdOx1 nCoV-19 in Common Variable Immunodeficiency

**DOI:** 10.3389/fimmu.2022.907125

**Published:** 2022-06-17

**Authors:** Vera Goda, Gergely Kriván, Andrea Kulcsár, Márton Gönczi, Szabolcs Tasnády, Zsolt Matula, Ginette Nagy, Gabriella Bekő, Máté Horváth, Ferenc Uher, Zoltán Szekanecz, István Vályi-Nagy

**Affiliations:** ^1^ Pediatric Hematology and Stem Cell Transplantation Department, Central Hospital of Southern Pest, National Institute of Hematology and Infectious Diseases, Budapest, Hungary; ^2^ Department of Special Immunization Services, Central Hospital of Southern Pest, National Institute of Hematology and Infectious Diseases, Budapest, Hungary; ^3^ Central Laboratory of Central Hospital of Southern Pest, National Institute of Hematology and Infectious Diseases, Budapest, Hungary; ^4^ Laboratory for Experimental Cell Therapy, Central Hospital of Southern Pest, National Institute of Hematology and Infectious Diseases, Budapest, Hungary; ^5^ Departmental Group of Infectious Diseases, Semmelweis University Doctoral School of Clinical Medicine, Budapest, Hungary; ^6^ Department of Rheumatology, Faculty of Medicine, University of Debrecen, Debrecen, Hungary; ^7^ Department of Hematology and Stem Cell Transplantation, Central Hospital of Southern Pest, National Institute of Hematology and Infectious Diseases, Budapest, Hungary

**Keywords:** common variable immunodeficiency (CVID), primary immunodeficiency, anti-SARS-CoV-2 antibodies, IFN-γ producing T cells, IFN-γ ELISpot assay, vaccination

## Abstract

Common variable immunodeficiency (CVID) patients have markedly decreased immune response to vaccinations. In this study we evaluated humoral and T cell-mediated responses against severe acute respiratory syndrome coronavirus-2 (SARS-Cov-2) with additional flow cytometric changes in CVID patients receiving booster vaccination with BNT162b2 after two ChAdOx1 nCoV-19. The BNT162b2 vaccine raised the anti-spike protein S immunoglobulin G over the cut-off value from 70% to 83% in CVID, anti-neutralizing antibody had been raised over a cut-off value from 70% to 80% but levels after boosting were significantly less in both tests than in healthy controls (*p=0.02; **p=0.009 respectively). Anti-SARS-CoV-2 immunoglobulin A became less positive in CVID after boosting, but the difference was not significant. The cumulative interferon-γ positive T cell response by ELISpot was over the cut-off value in 53% of the tested individuals and raised to 83% after boosting. This and flow cytometric control of cumulative CD4+ and CD8+ virus-specific T cell absolute counts in CVID were also statistically not different from healthy individuals after boosting. Additional flow cytometric measures for CD45+ lymphocytes, CD3+, and CD19+ cells have not shown significant differences from controls except for lower CD4+T cell counts at both time points (**p=0.003; **p=0.002), in parallel CD4+ virus-specific T-cell ratio was significantly lower in CVID patients at the first time point (*p: 0.03). After boosting, in more than 33% of both CVID patients and also in their healthy controls we detected a decrease in absolute CD45+, CD3+, CD3+CD4+, and CD3+CD8+, CD19+, and CD16+56+ cell counts. CD16+CD56+ cell counts were significantly lower compared to controls before and after boosting (*p=0.02, *p=0.02). CVID patients receiving immunosuppressive therapy throughout the previous year or autologous stem cell transplantation two years before vaccination had worse responses in anti-spike, anti-neutralizing antibody, CD3+CD4+T, CD19+ B, and natural killer cell counts than the whole CVID group. Vaccinations had few side effects. Based on these data, CVID patients receiving booster vaccination with BNT162b2 after two ChadOx1 can effectively elevate the levels of protection against COVID-19 infection, but the duration of the immune response together with COVID-19 morbidity data needs further investigation among these patients.

## Introduction

Severe acute respiratory syndrome coronavirus-2 (SARS-CoV-2), which emerged in 2019, continues to cause significant morbidity and mortality worldwide (1). When assessing the risk factors for severe SARS COVID-2 infection primary immunodeficiency is among the important host factors (1). Common Variable Immunodeficiency (CVID) is the most frequent inborn error of immunity (IEI) characterized by decreased humoral immune responses and being the model of primary antibody deficiencies (PAD). CVID usually presents with recurrent or severe infections (1, 2) and a notable minority also has autoimmune sequelae or malignant lymphoproliferative diseases that might necessitate immunosuppressive treatments. These therapies -including B cell depleting drugs, glucocorticoids, mycophenolate, abatacept, and multidrug immunosuppressive treatments - are also associated with lower vaccine response rates (3). Patients with the above-mentioned comorbidities and immunosuppressive therapies have common abnormalities in T cell numbers or function, the decreased total CD4+ T cell count is associated with recurrent infections, the decrease of naive CD4+T cell or natural killer (NK) cell ratio is associated with autoimmunity and decreased NK cell count in CVID patients seemed to have no association with viral infections (4, 5). The majority of CVID patients have normal numbers of B cells but some have low or absent B cells that can contribute to decreased vaccine responses. Primary antibody deficient patients receive regular immunoglobulin replacement therapy (1, 2). By the end of last year, many manufacturers declared that subcutaneous immunoglobulin (SCIG) or intravenous immunoglobulin (IVIG) might contain a considerable quantity of antibodies against SARS-CoV-2 infection that can not be separated when measuring antibodies. Observing these phenotypic characteristics in CVID we can conclude the complexity of immune response against infections and vaccinations. Studies in healthy individuals showed that effective T-cell response is associated with milder COVID-19 disease (6). Circulating SARS-CoV-2 specific CD4+ T cell response against spike protein was correlated with the magnitude of anti-SARS-CoV-2-IgG, IgA titers (7). More interestingly, T cells against closely related SARS-CoV-1 virus had been detected as long as 11 years after the recovery, whereas no antigen-specific memory B cells or antibodies had been detected years after the infection (8). Regarding immunogenicity of anti-SARS-CoV-2 vaccines in CVID, most of the studies were done on a limited number of patients receiving mostly homologous BNT162b2 immunization and evaluated immune responses after the second but not the third dose (3, 9–19). Following severe adverse reactions to the ChAdOx1 Oxford/Astra Zeneca, European health authorities permitted the switch to the second dose with the BNT162b2 vaccine. Later, a real-world observational study of health care workers showed that heterologous ChAdOx1/BNT162b2 combination can induce better protection against SARS-CoV-2 infection than homologous BNT162b2/BNT162b2 combination. Sera from heterologous vaccinated individuals displayed a stronger neutralizing activity, regardless of the SARS-CoV-2 variant. This enhanced neutralizing potential was correlated with increased frequencies of switched and activated memory B cells recognizing the SARS-CoV-2 Receptor Binding Domain (RBD). ChAdOx1 induced a weaker IgG response but a stronger T cell response than the BNT162b2 vaccine after the priming dose, which could explain the complementarity of both vaccines when used in combination (20, 21). A heterologous vaccination regimen could therefore be particularly suitable for immunocompromised individuals. In spring 2021, we only had the opportunity to vaccinate our PAD patients frontline with ChAdOx1 in our center, so we decided to start mass vaccination of our patients and in autumn we administered the third vaccine as a booster with BNT162b2. We aimed to detect humoral and cellular immune responses after heterologous vaccination among primary immunodeficiency patients at their third vaccination with BNT162b2.

## Materials and Methods

We used serological assays, flow cytometry measures for lymphocytes, T, B, NK cell counts T cell subsets, and virus-specific T cell count against SARS-CoV-2, as well as T cell ELISpot technology to detect IFN-γ release from immune cells after exposure to SARS-CoV-2 spike (S1 and S2), nucleocapsid (N), membrane (M), and envelop (E) peptide. We assessed SARS-CoV-2-specific antibody and T cell responses in CVID patients with a mean time of 146 days from the second dose of ChadOX1 on the day of BNT162b2 vaccination, the same vaccine interval was 147 days in HC. The mean dose interval between the two ChAdOx1 doses was 50 days in CVID and 75 days in HC.

### Patients

In our center, we started to administer ChAdOx1 to the first 70 patients with CVID in March. The selection of the patients was in the order of their registration. We collected the data of 30 of our patients with CVID immunized with ChadOx1, followed by a booster vaccination with BNT162b2 in our center. Specimens were collected including a group of 10 patients - called the “CVID plus” group-, who had hematologic malignancy or autoimmune disease necessitating immunosuppressive therapy throughout the year before the first vaccination or an autologous hematologic stem cell transplantation (auto-HSCT) carried out two years before the first vaccination against SARS-CoV-2. The healthy control group (HC) consisted of 15 volunteers with no immunological or hematological deviation to our knowledge. Specimens were obtained between 09. 09. 2021 and 11. 11. 2021 at two time points: the first was on the day prior, and the second was on the 14^th^ day of the third vaccination. Exclusion criteria were a known history of COVID-19, having fever, upper respiratory infectious symptoms, cough, or diarrhea 10 days before vaccinations. Polymerase chain reaction (PCR) to detect SARS-CoV-2 infection was performed in case of possible signs or symptoms of SARS-CoV-2 infection. Written informed consent was obtained from all participants before enrollment.

### Humoral Response Detection Methods

Venous blood was collected in vacutainer tubes and serum samples. SARS-CoV-2 antibodies were detected using commercially available test systems such as 1, LIAISON XL^®^ SARS-CoV-2 S1/S2 IgG test CLIA (DIASORIN S.P.A., Saluggia, Italy) detecting anti-S1/S2 IgG antibodies, 2, SARS-CoV-2 Surrogate Virus Neutralization Test Kit (GenScript Biotech B. V., Leiden, Netherlands) measuring IgG levels against SARS-CoV-2 RBD by ELISA, 3, SARS CoV-2 NP IgG CMIA Architect (Abbott Laboratories, Abbott Park, IL, USA) detecting SARS-CoV-2 nucleocapsid-specific (NP) IgG, and 4, SARS-CoV-2 S IgA ELISA(EUROIMMUN Medizinische Labordiagnostika AG, Lübeck, Germany) measuring IgA levels against SARS-CoV-2 spike protein.

### T-Cell Response Detection Methods

To evaluate SARS-CoV-2 virus-specific T (VST) cell immunity in vaccinated individuals we used ELISpot measurements. We used freshly isolated peripheral blood mononuclear cells (PBMC) isolated by density gradient centrifugation using the Leucosep Kit (Oxford Immunotec Ltd, Abingdon, Oxfordshire, UK). Altogether 250,000 recovered PBMCs were plated into each well of a TSPOT ^®^ Discovery SARS-CoV-2 (Oxford Immunotec) kit that quantifies IFN γ-producing T cells in response to viral peptides. The kit is composed of five different but overlapping peptide pools to cover protein sequences of five different SARS-CoV-2 antigens including S1, S2, N, M, and Env. Peptides that showed high sequence homology to endemic coronaviruses were removed from the peptide pools by the manufacturer. The cumulative spot forming units (SFU) per 2.5x105 PBMC of individuals was calculated as the sum of T-SPOTs for S1, S2, N, M, and E antigens minus the background. We also used a functional flow cytometry-based assay from blood samples taken in BD Vacutainer^®^ CPT™ Mononuclear Cell Preparation tubes. After the gradient separation of the mononuclear cells (MNCs), we used peptide pools of the SARS-CoV2 virus to stimulate IFN-γ production of the T cells (SARS-CoV2-SELECT peptivator Miltenyi Biotec Bergisch Gladbach, Germany) according to the manufacturer’s guide. After 6 hours of stimulation, we detected the IFN-γ producing T cells with the Rapid Cytokine Inspector CD4-CD8 T Cell Kit (Miltenyi Biotec Bergisch Gladbach, Germany), following the manufacturer’s instructions. For data acquisition and analysis we used a BD FACS CANTO II flow cytometer with the DIVA software (BD Biosciences). By the use of a sequential gating strategy, the percentage of IFN-γ producing cells has been identified within the CD4+ and CD8+ populations.

### Lymphocyte Immunophenotyping Methods

To measure lymphocyte subpopulations, the 6-color TBNK kit (BD Biosciences, San Jose, USA) was used, according to the manufacturer’s guide. The samples were EDTA coagulated whole blood samples. The kit uses a lyse-no-wash staining procedure, to give absolute cell numbers, we used the single platform method with BD Trucount tubes. For data acquisition and analysis we used a BD FACS CANTO II flow cytometer with the DIVA software (BD Biosciences).

### Statistical Analysis

All statistical analyses to compare the results of patients and HC were performed applying two-tailed t-probe and f-probe, Pearson test, Chi-squared test as appropriate. *p<0.05 was considered to be statistically significant. Descriptive statistical analyses such as median and range were calculated using non-transformed data. Positive and negative cut-off values were adopted from the manufacturer’s package inserts. Data availability: All data, materials, and methods used in the analysis will be available from the corresponding author upon request.

## Results

### Patient Characteristics

Patient characteristics focusing on clinical presentations, particularly hematological malignancies, and autoimmune diseases necessitating immunosuppressive therapy can be observed in **Table 1**. The average patient age was 44 years in the CVID group, and 50 years in the HC group. The male-to-female ratio was 1:1 in CVID, and 1,14:1 in HC. All patients with common variable immunodeficiency fulfilled the European Society for Immunodeficiencies 2019 criteria for diagnosis of probable CVID (22). All of the patients were on immunoglobulin replacement therapy, 6 on SCIG, and 24 on IVIG supply. Patients received their COVID-19 vaccine with a mean interval of 14 days apart from their next IVIG infusion date. The vaccines were generally well tolerated with limited injection site pain being the most common reported adverse event. Local reactions were pain, redness, swelling, or axillary lymphadenopathy; while fever, headache, weakness, or fatigue limiting normal activity were considered systemic side effects. Among the CVID patients, 53.3% (16/30) had only local reactions in contrast to the HC group with 26.6% (4/15) highlighting a phenomenon: CVID patient who usually has a worse immune response to vaccinations might have a better profile of adverse events with a less systemic reaction but the difference was not significant (*p=0.09). Adverse events were similar to those previously described (3), none of the patients reported long-lasting adverse events. During the study, participants were not tested for active infection with virus polymerase chain reaction (PCR) assay, only in case of possible signs of infection. By the time this manuscript was written only three CVID patients contracted SARS-CoV-2 infection and all of them were at least 2 weeks from the time of their booster vaccination. Nobody from the HC group has had a COVID-19 infection so far. One CVID female patient had mild disease. The other female was hospitalized for weakness, fluid imbalance, and high fever. She was treated with remdesivir for 10 days and was later discharged. The worst case of the “CVID plus” group is a male patient suspected to have fast-growing mediastinal lymphoma under investigation. He had been hospitalized, and COVID 19-specific ground-glass opacity was seen on chest computed tomography that resolved in three weeks. Histology confirmed non-Hodgkin lymphoma but he was admitted to the intensive care unit after the thoracic biopsy was on mechanical ventilation but after initial immunosuppressive therapy was successfully extubated.

**Table 1:** CVID patient’s characteristics.

Patient N.	Age (y)	Gender	Clinical manifestations, concomitant diseases	Last ISU treatment or HSCT
**CVID patients without hematologic malignancy or active autoimmune disease not on immunosuppressive therapy**				
I/1	49,575	f		
I/2	26,622	f		
I/3	43,019	f		
I/4	59,178	f		
I/5	51,121	m		
I/6	22,521	m		
I/7	26,507	f	enteropathy	
I/8	21,044	f	granuloma	
I/9	60,197	m	coeliakia, emphysema, chr bronchitis, hepatopathy	
I/10	35,288	m		
I/11	41,753	m	Dupuytren's contracture, epicondylitis lat. humeri	
I/12	58,863	m		
I/13	38,997	m	IDDM	
I/14	27,271	m	Hypertonia, thyroiditis, GERD, obesity, sleep apnoea	
I/15	46,482	f	TIA	
I/16	43,121	m	Crohn disease	last ISU Th.: 2012
I/17	71,077	m	Wegener gr. in remission	last ISU Th.: 2019
I/18	32,729	f	enteropathy	
I/19	34,504	f	familiar CVID, Ewing sc 1997, poliallergy, salicylate allergy	last ISU Th. :1997
I/20	49,299	f	RA	last ISU Th.: 2018
**CVID plus patients with hematologic malignancy or active autoimmune disease necessitating ISU or HSCT**				
II/1	42,696	f	aspec. colitis, B12 deficiency, atrophic gastritis, scleroderma	on tocilizumab, Mtx, low dose MP
II/2	60,638	f	Waldenström MGUS (2014), CVID dg 2004, seroneg RA	on tocilizumab
II/3	65,378	f	familial CVID, T-cell NHL, thrombocytopenia	splenectomy, last ISU Th.:2018
II/4	49,844	f	ITP, Crohn’s disease, SNSA enthesopathy	last ISU Th.: Jun 2021
II/5	67,321	m	CIDP, low-grade B cell lymphoma, epilepsy, Hbs ag pos.	last ISU Th.: 2021
II/6	57,416	m	Wegener gr., emphysema, pulmonary embolism	on MP
II/7	42,173	m	NHL	autologous HSCT 2019
II/8	41,545	f	APS, ITP, RA, epilepsy	on CSA
II/9	30	m	PID+lymphoma ly predominant HL 2020	no ISU Th
II/10	44,586	m	lymphoma, chr RF, nephrolithiasis, hypothyreosis	splenectomy

The first 20 patient (I/1-20) is in remission of autoimmune diseases or quit immunosuppressive therapy one year before their first vaccination. The last 10 patient is the “CVID plus” subgroup (II/1-10) who has malignancy or autoimmune disease necessitating immunosuppressive therapy in the year before the start of their anti-COVID-19 vaccination or had autologous HSCT two years before the same vaccination.

CVID, common variable immunodeficiency; ISU Th, immunosuppressive therapy; HSCT, hematopoietic stem cell transplantation; MP, methylprednisolone; IDDM, insulin-dependent diabetes mellitus, GERD, gastroesophageal reflux disease; TIA, transitory ischemic attack; Wegener gr, Wegener granulomatosis; MGUS, monoclonal gammopathy of undetermined significance; RA, rheumatoid arthritis; NHL, non-Hodgkin lymphoma; HBs ag pos, hepatitis B antigen positive; APS, Antiphospholipid syndrome; ITP, immune thrombopenia; HL, Hodgkin lymphoma; CRF, chronic renal failure.

### Humoral Immune Response to Vaccination

**Figure 1:**
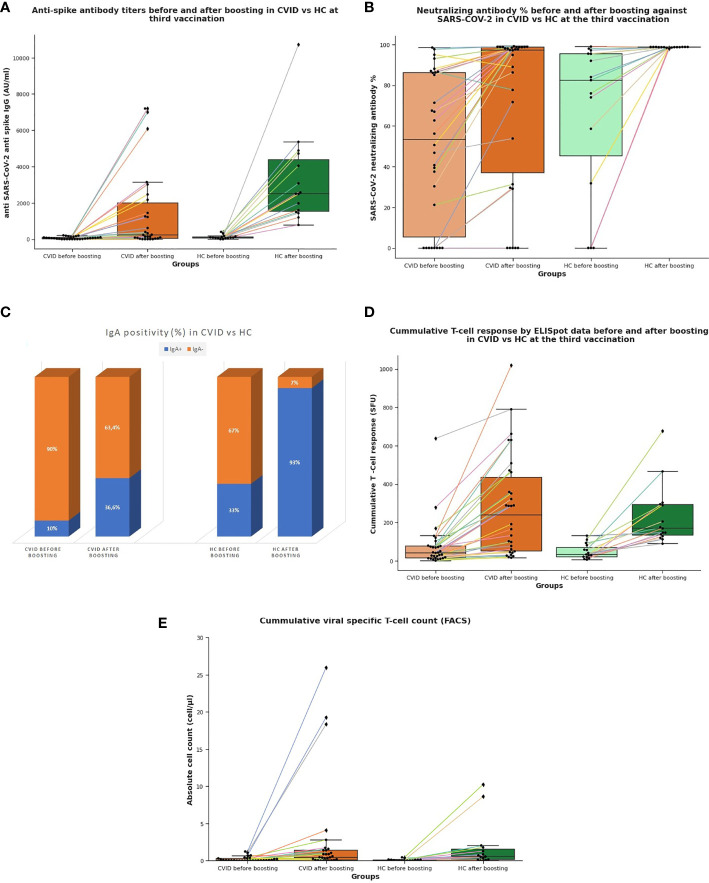
**(A)** Anti-SARS-CoV-2 spike immunoglobulin G levels (AU/ml) after boosting in CVID is significantly lower compared to HC at the third vaccination. **(B)** Neutralizing antibody response (%) against SARS-CoV-2 had been raised close to a 100% after boosting at the third vaccination in healthy controls while the response in CVID had a broad distribution. **(C)** IgA positivity raised from 10% to 36.6% in CVID and from 33% to 93% among HC. **(D)** Cumulative T cell response (SFU) by ELISpot is significantly not different at the third vaccination in CVID from HC before and after boosting. **(E)** Cumulative viral specific T cell count by flow cytometry is significantly not different at third vaccination in CVID from HC before and after boosting. CVID, common variable immunodeficiency; HC, healthy control; SARS-CoV-2, severe acute respiratory infection coronavirus 2; SFU, spot forming unit.

BNT162b2 vaccine raised the anti-spike protein (S) IgG (DiaSorin) over the cut-off (15 AU/ml) from 70% to 83% in CVID compared to the rise from 87% to 100% in HC. After boosting, CVID patients had a significantly lower anti-spike protein elevation than HC (*p=0.02473), the difference was lower in the CVID plus group (*p=0.04). Data can be seen in **Figure 1A**. Neutralizing antibody had been raised over a cut-off value (30%) from 70% to 80% but levels after boosting were significantly less than control levels, which were all above the cut-off value at both time points (**p=0.009), the difference was even stronger in CVID plus group (**p=0.002). Data can be seen in **Figure 1B**. Anti-SARS-CoV-2 IgM positivity raised from 10 to 16.6% in CVID patients, HC control positivity rise was also limited from 6.6% to 20%; Anti-SARS-CoV-2 NP IgG was negative in both groups at both time points correlating with no prior COVID-19 clinical infection. Anti-SARS COVID-2 IgA became less positive in CVID after boosting than in healthy controls but the change was statistically not significant (p=0.07), IgA positivity raised from 10% to 36.6% in CVID and from 33% to 93% among HC **Figure 1C**.

### T Cell Response Following Vaccination

The magnitude of cumulative T cell response was not significantly higher among healthy controls than in CVID or “CVID plus” group at any time point (p=0.26 and p=0.43 for the CVID group; p=0.38 and p=0.49 for the CVID plus group), data can be seen in **Figure 1D**. After the third vaccination of CVID patients and their HC, both had some response at least to one of the peptide pools. ELISpot assay was over the cut-off value (40 SFU) in 53% of CVID patients before the booster shot raising to 83% at the second time point while HC raised from 46.6% to 100% after boosting. In parallel, we measured the cumulative CD4+ or CD8+ VST cell ratio, at least one of them had been elevated over the cut-off value from 56.6% to 79.3% in CVID while it raised from 60% to 93.3% in HC after boosting. The cut-off value was the 0.01% cumulative CD4+ or CD8+ VST cell ratio being eligible for plasma therapy in our center **Figure 1E**. There was no significant difference in cumulative CD3+ CD4+ VST and CD3+CD8+ VST absolute counts compared to HC at both time points in CVID (p=0.1 and p=0.47). Coronavirus-specific CD3+CD4+ T cell ratio alone was significantly lower in CVID before boosting than in HC (*p=0.033). CD3+CD8+ VST cell ratio was not significantly lower than HC alone. One patient’s VST results were elusive because of the aggregation of cells at the second time point. Looking for correlation with CD3+CD8+VST cell absolute count with Pearson test ELISpot showed a coefficient magnitude of 0.620413 and 0.655033 in CVID and HC groups respectively indicating a moderately strong relationship.

### Lymphocyte Phenotyping by Flow Cytometry

**Figure 2:**
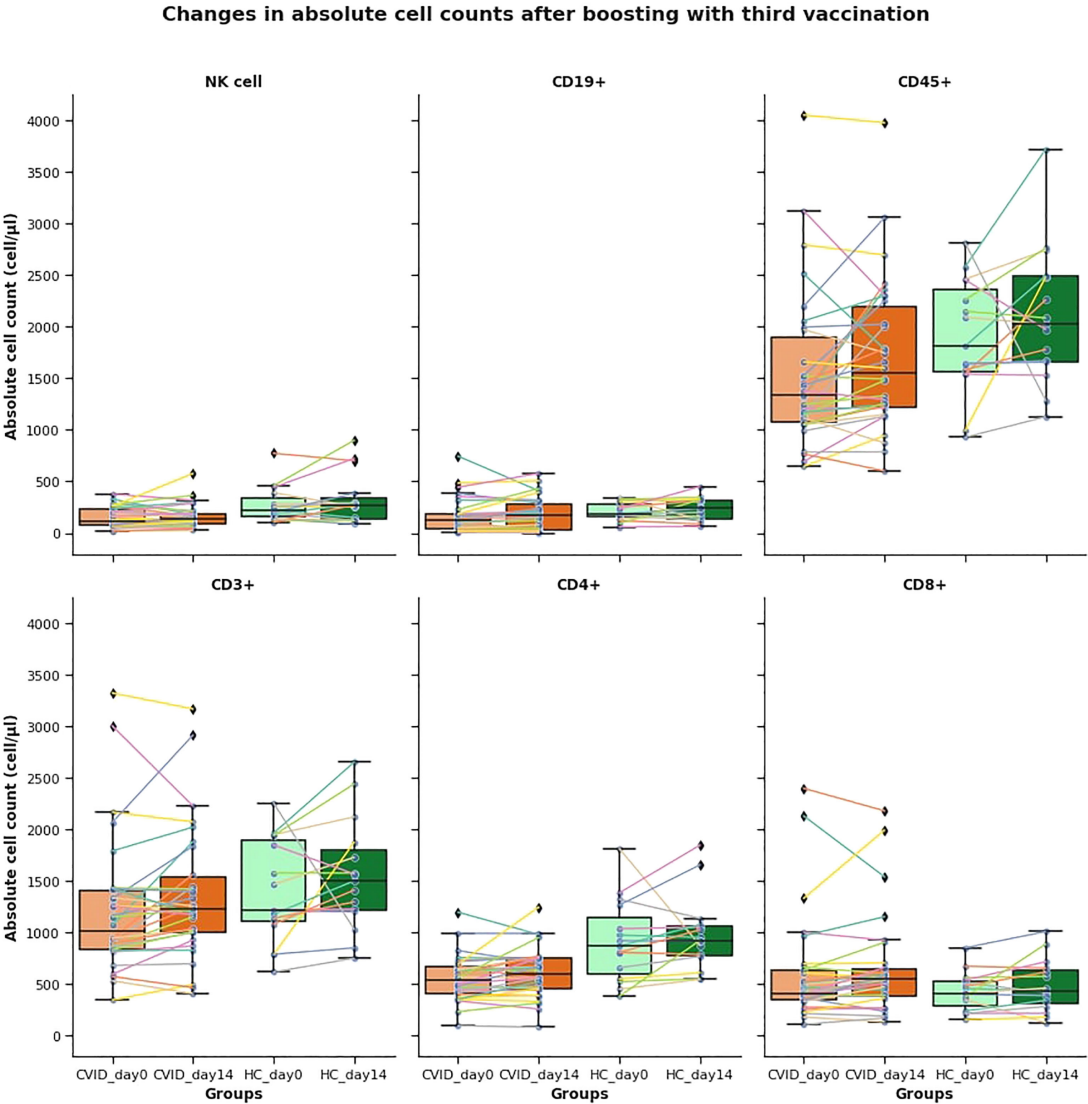
Mean changes in absolute cell counts after boosting at third vaccination. More than 33% of patients and healthy controls have decreased lymphocyte and lymphocyte subpopulation absolute cell counts, therefore we divided the patients and their HC to further subgroups depending on decrease or elevation and illustrated the mean changes of absolute lymphocyte counts in the different subgroups. Mean counts of decreased cell counts were not statistically different in CVID from healthy controls.

For analyses of main lymphocyte subsets in vaccinated individuals, we defined the absolute numbers of CD45+, CD3+ T, CD3+CD4+T, and CD3+CD8+ T lymphocytes, CD4+/CD8+ T cell ratio, CD19+ B, and CD3-CD16+56+ NK cells in their blood samples by flow cytometry. CD16+CD56+, CD3+CD4+ cell counts and the CD4+/CD8+ T cell ratio were significantly lower in CVID and CVID plus group at both time points compared to HC (CD16+CD56+ [(*p=0.017 and *p=0.023 for CVID group, *p=0.026 and *p=0.032 for CVID plus group)]; CD3+CD4+ [**p=0.004 and **p=0.002 for CVID group, ***p=0.0004 and ***p=0.00008 for CVID plus group)]; CD4+/CD8+ ratio [(**p= 0.002 and **p=0.005 for CVID, **p=0.001 and **p=0.002 for CVID plus group)] respectively). CD19+ B cell counts were significantly lower only in the CVID plus group at both time points (*p=0.041 and *p=0.043). We did not find statistically significant differences in CD3+CD8+ T cell counts in CVID patients compared to HC at any time point (CD3+CD8+ T p=0.12 and p=0.14). More than 33% of patients and also their healthy controls have decreased lymphocyte and lymphocyte subpopulation absolute cell counts (CD45+, CD3+, CD3+CD4+, CD3+CD8+, CD19+, CD16+CD56+), therefore we divided the patients and their HC to further subgroups depending on decrease or elevation and illustrated the mean changes of absolute lymphocyte counts in the different subgroups to represent its extent in **Figure 2**. Mean changes in absolute cell counts were not statistically different in CVID from healthy controls except for NK cells in the elevated group, which was significantly lower than HC (*p=0,049). We did not find any strong correlation between the above-mentioned decreased parameters and the anti-spike IgG or T cell responses in any group. Those CVID patients, whose CD45+, CD3+, or CD3+CD8+ counts did not decrease, tended to have a better antibody response based on anti-spike IgG elevation. This observation has not been reported in the literature and there is no information provided in the description of the BNT162b2 EMA Assessment Report.

## Discussion

People living with primary immunodeficiencies are more susceptible to SARS-CoV-2 infection and had higher hospitalization necessities and mortality rates compared to healthy individuals before vaccination against coronavirus infection. At the beginning of 2021, it was crucial to vaccinate these people as soon as possible and evaluate their immune response to establish a long-term vaccination protocol. Published data on humoral and cellular responses among primary immunodeficient patients determined different results after homologous vaccination mainly with BNT162b2 and solely measured after the second dose. Kinoshita et al. (2021) described the first 5 PAD patients having a robust humoral and cellular response after BNT162b2 similar to healthy controls (16). In a small cohort of patients with IEI, 18 of 22 (81.8%) tested positive for anti-S IgG antibodies and 19 of 26 patients (73.1%) showed a T cell response (13). In the next study, 11 from 15 (73.33%) CVID patients had protective levels of SARS-CoV-2 S1 IgG with a wide range of titers (17). After homologous vaccination of 18 patients, vaccinated mainly with mRNA vaccines, both humoral and cellular responses were lower in CVID but 83% had S1-specific antibody response and 83% had S1-specific T cells compared with the results of healthy controls (HC), lower responses were associated with former autoimmunity, lymphoproliferation or B cell depletion therapies (9). In PAD patients other studies emphasized low BNT162b2 induced memory B-cell response and much lower antibody responses than the former ones (12, 14, 15). In our observational study, assuming that CVID patients might benefit from heterologous vaccination, we compared the antibody and T cell responses to SARS-CoV-2 vaccination in 30 CVID patients and 15 healthy controls of boosting with BNT162b2 after being primed with two ChAdOx1 vaccination. Some of the parameters were analyzed in a subgroup of CVID patients called the “CVID plus” group having autoimmune or lymphoproliferative disease necessitating recent immunosuppressive therapy or auto-HSCT described above. Timing of IVIG supply for CVID patients might be of importance to provide maximum effectiveness, especially now, when anti-SARS-CoV-2 antibody content in IVIG products is increasing. By some recent guidelines (ASCIA guideline, Leeds Teaching Hospitals NHS Trust), we also advised our patients to take their third vaccination after 14 days of previous IVIG supplementation, taking an average of 14 days from previous IVIG supplementation to the vaccination. We collected clinical data on vaccination side effects in the CVID group and found less remarkable systemic side effects compared to the control individuals, but the significance is low. In our study, we demonstrated a further elevation in humoral responses compared with the second dose of COVID-19 vaccination data. BNT162b2 vaccine raised the anti-spike IgG over the cut-off to 83% in CVID, Neutralizing antibodies had been raised over the cut-off value in 80% of patients, but they had a significantly lower anti-spike protein elevation than HC and the difference was even more significant in the “CVID plus” group. Anti-SARS-CoV-2 IgM had a limited value in clinical practice. Anti-SARS-CoV-2 NP was negative in the patient and control group at both time points and we did not have detected COVID-19 infection before vaccination. Anti-SARS COVID-2 IgA became less positive in CVID after boosting than healthy controls but the difference was not significant. The magnitude of cumulative T cell response was not significantly higher among healthy controls than in CVID or CVID plus group even after three months from their second ChAdOx1 vaccination or after boosting with BNT162b2. ELISpot assay for T cell response was over the cut-off value in 53% of CVID patients before and rose to 83% after the booster. In parallel, there was no significant difference in cumulative coronavirus-specific CD3+ CD4+ and CD8+ absolute counts after boosting compared to HC. Looking for correlation with virus-specific CD3+CD8+T cell absolute count with Pearson test ELISpot showed a coefficient magnitude of 0.62 and 0.65 in CVID and HC groups respectively, indicating a moderately strong relationship. Lymphocyte, T, B, and NK cell, CD3+CD4+, and CD3+ CD8+ cell subset counts were decreased at least in 33% of our patients, and their healthy controls after boosting with considerable mean changes in absolute counts. This observation has not been documented before in the literature. In those CVID patients, whose CD45+, CD3+, or CD3+CD8+ counts were elevated, we had the observation of better antibody response based on anti-spike IgG elevation. CD16+CD56+ cells, CD3+CD4+ cell counts were significantly lower in CVID and “CVID plus” group at both time points than in HC which might also have a role in vaccine efficacy and needs further evaluation. CD19+ B cell counts were significantly lower in the CVID plus group at both time points but not in the whole patient group accordant with the suspected deeper antibody deficient state. By the time this manuscript was written only three CVID patients contracted SARS-CoV-2 infection and all of them were at least 2 weeks from the time of their booster vaccination. Taken together all of these cases might not weaken the potential protection of vaccination against coronavirus infection. Limitations of our study are the low number of patients but former studies had even less uniform parameters like age distribution, diversity of PAD/IEI patients, IVIG supplementation schedule, and type of vaccines. In this paper, we could not include the results of the same disease group with homologous BNT162b2 vaccination but propose later to compare it with our heterologous vaccination results. Further limitations are that the PCR test to detect SARS-CoV-2 infection was only performed in case of a sign of possible SARS-CoV-2 infection and that the elapsed time is very short from booster vaccination. Preliminary data like the OCTAVE Trial (P Kearns et al. UK) show that the fourth dose is necessary for immunocompromised patients (23). Most but not all of our patients already got the fourth vaccination against SARS-CoV-2 infection, therefore the efficacy of the first three vaccines alone cannot be determined later. To our best knowledge, our work represents the first comparative analysis of adaptive immunity in a cohort of CVID patients compared to healthy controls receiving heterologous vaccination priming with two ChAdOx1 vaccines followed by a BNT162b2 booster. Based on these data, we can conclude that for patients living with CVID the above described heterologous vaccination protocol was immunologically effective but humoral responses were significantly lower than in healthy controls. However, the duration of the third vaccination-induced immunological response, COVID-19 morbidity data need further investigation.

## Data Availability Statement

The original contributions presented in the study are included in the article/supplementary material. Further inquiries can be directed to the corresponding author.

## Ethics Statement 

The studies involving human participants were reviewed and approved by Institutional Review Board of the Central Hospital of Southern Pest - National Institute of Hematology and Infectious Diseases. (Registration number 2397-001/2022). The patients/participants provided their written informed consent to participate in this study.

## Author Contributions

VG, IV-N, and GB designed the study. FU, MG, ST, ZM, and GN performed experiments. VG and MH conducted data analyses. GK and VG conducted participant interviews, enrollment, and specimen collection. VG and AK organized and performed vaccinations and clinical part of data collection. IV-N and ZS performed supervision. VG drafted the manuscript. All authors contributed to the article and approved the submitted version.

## Funding

This work was supported by the National Research, Development, and Innovation Office (Grant Number: 2020-1.1.6-JÖVŐ-2021-00011) and the National Public Health Center (Grant Number: EFOP-1.8.0-VEKOP-17-2017-00001). Emberi Erőforrások Minisztériuma 2022 250 Million Project.

## Conflict of Interest

The authors declare that the research was conducted in the absence of any commercial or financial relationships that could be construed as a potential conflict of interest.

## Publisher’s Note

All claims expressed in this article are solely those of the authors and do not necessarily represent those of their affiliated organizations, or those of the publisher, the editors and the reviewers. Any product that may be evaluated in this article, or claim that may be made by its manufacturer, is not guaranteed or endorsed by the publisher.

## Abbreviations

APS, Antiphospholipid syndrome; ASCIA, Australian Society of Clinical Immunology and Allergology; BBIBP-CorV, Beijing Bio-Institute of Biological Products Coronavirus Vaccine; BNT162b2, Pfizer-BioNTech vaccine; CD, a cluster of differentiation; CD40L, a cluster of determination 40 ligand; ChAdOx1, Oxford/Astra Zeneca vaccine (also named as ChAdOx1 nCoV-19 or AZD1222); CLIA, Clinical Laboratory Improvement Amendments; COVID-19, coronavirus 19; CRF, chronic renal failure; CVID, common variable immunodeficiency; ELISA, enzyme-linked immunosorbent assays; ELISpot, enzyme-linked immune absorbent spot; GERD, gastroesophageal reflux disease; HBs ag pos, hepatitis B antigen positive; HC, healthy control; HL, Hodgkin lymphoma; HSCT, hematopoietic stem cell transplantation; IDDM, insulin-dependent diabetes mellitus; IEI, Inborn Errors of Immunity; IFN-γ, interferon gamma; ISU Th, immunosuppressive therapy; ITP, immune thrombopenia; IVIG, intravenous immunoglobulin; MGUS, monoclonal gammopathy of undetermined significance; MNCs, mononuclear cells; MP, methylprednisolone; mRNA-1273, messenger ribonucleic acid- 1273 vaccine; NHL, non-Hodgkin lymphoma; NHS, National Health System; NK, Natural Killer; PAD, primary antibody deficiencies; PBMC, peripheral blood mononuclear cell; PCR, polymerase chain reaction; RA, rheumatoid arthritis; RBD, receptor binding domain; SARS-CoV-2, severe acute respiratory infection coronavirus 2; SCIG, subcutaneous immunoglobulin; s VNT Kit, Surrogate Virus Neutralization Test Kit; TIA, transitory ischemic attack; VST, virus-specific T cell; Wegener gr, Wegener granulomatosis.
